# An Integrated Arterial Remodeling Hydrogel for Preventing Restenosis After Angioplasty

**DOI:** 10.1002/advs.202307063

**Published:** 2024-02-11

**Authors:** Chenxing Fu, Qiu Li, Minghui Li, Jiexin Zhang, Feiran Zhou, Zechuan Li, Dongyue He, Xinyi Hu, Xiaodong Ning, Wenjie Guo, Weirun Li, Jing Ma, Guoqin Chen, Yafang Xiao, Caiwen Ou, Weisheng Guo

**Affiliations:** ^1^ Department of Minimally Invasive Interventional Radiology The Second Affiliated Hospital School of Biomedical Engineering Guangzhou Medical University Guangzhou 510260 China; ^2^ Department of Cardiology Laboratory of Heart Center Zhujiang Hospital Southern Medical University Guangzhou 510280 China; ^3^ Department of Cardiology Panyu Central Hospital Guangzhou University of Chinese Medicine Guangzhou 510006 China

**Keywords:** angioplasty, curcumin, nitric oxide, peptide hydrogel, self‐assembly, vascular remodeling

## Abstract

The high incidence of restenosis after angioplasty has been the leading reason for the recurrence of coronary heart disease, substantially increasing the mortality risk for patients. However, current anti‐stenosis drug‐eluting stents face challenges due to their limited functions and long‐term safety concerns, significantly compromising their therapeutic effect. Herein, a stent‐free anti‐stenosis drug coating (denoted as Cur‐NO‐Gel) based on a peptide hydrogel is proposed. This hydrogel is formed by assembling a nitric oxide (NO) donor‐peptide conjugate as a hydrogelator and encapsulating curcumin (Cur) during the assembly process. Cur‐NO‐Gel has the capability to release NO upon β‐galactosidase stimulation and gradually release Cur through hydrogel hydrolysis. The in vitro experiments confirmed that Cur‐NO‐Gel protects vascular endothelial cells against oxidative stress injury, inhibits cellular activation of vascular smooth muscle cells, and suppresses adventitial fibroblasts. Moreover, periadventitial administration of Cur‐NO‐Gel in the angioplasty model demonstrate its ability to inhibit vascular stenosis by promoting reendothelialization, suppressing neointima hyperplasia, and preventing constrictive remodeling. Therefore, the study provides proof of concept for designing a new generation of clinical drugs in angioplasty.

## Graphical abstract

Schematic description of Cur‐NO‐Gel used in this study. Periadventitial administration of Cur‐NO‐Gel with the capability for sustained Cur release and on‐demand NO liberation. The integrated hydrogel exerts a three‐pronged approach to overcome the stenosis after angioplasty.

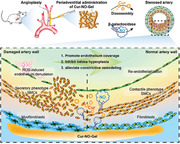

## 1 Introduction

Coronary heart disease, caused by pathological stenosis of the coronary artery, is presently the leading cause of mortality.^[^ ^1^ ^]^ Angioplasty is a widely employed approach in clinical practice for reestablishing blood flow to ischemic myocardium, with more than two million applications worldwide each year.^[^ ^2^ ^]^ However, the inevitable injury to the artery wall during the angioplasty procedure leads to a high incidence of restenosis, posing significant health risks to the postoperative patients.^[^ ^3^ ^]^ Neointimal hyperplasia, fueled by oxidative stress and excessive cytokines, drives the proliferation and migration of vascular smooth muscle cells (VSMCs) into the intima, serving as a primary cause of coronary restenosis. Moreover, the denuded endothelium resulting from the arterial injury continuously exposes VSMCs to cytokines and growth factors, exacerbating neointimal hyperplasia.^[^ ^4^ ^]^ Therefore, alongside direct neointimal cells elimination, promoting reendothelialization emerges as a promising strategy for reducing neointimal hyperplasia post‐angioplasty. Another significant contributor to coronary restenosis is the constrictive remodeling of the injured artery. This remodeling arises from angioplasty‐induced myofibroblast hyperactivity and excessive collagen matrix deposition in the adventitia. It manifests as artery shrinkage and subsequent luminal stenosis, becoming apparent 1–6 months after angioplasty.^[^ ^5^ ^]^ Currently, drug‐eluting stents represent the most widely used strategy in combating angioplasty‐induced restenosis. The coating drugs like paclitaxel and rapamycin can suppress neointimal hyperplasia by inhibiting VSMC proliferation.^[^ ^6^ ^]^ However, these drugs bring chronic endothelial toxicity and impede vascular reendothelialization, resulting in 5%−10% relapse rates within five to ten years post‐angioplasty.^[^ ^7^ ^]^ Furthermore, although stent implantation can maintain arterial tension and prevent elastic recoil, in the case of balloon angioplasty without stent placement, it is challenging to avoid contraction remodeling induced by the outer and middle layers’ constriction.^[^ ^8^ ^]^ Therefore, there is a pressing need to design an integrated anti‐restenosis drug capable of inhibiting neointima hyperplasia, promoting reendothelialization, and alleviating constrictive remodeling in one entity.

Curcumin (Cur), a phytochemical compound derived from turmeric, has been historically acknowledged in traditional Indian medicine for its advantageous properties, including anti‐oxidation, anti‐inflammation, anti‐tumorigenicity, and cardio‐protection.^[^ ^9^ ^]^ Studies have demonstrated that Cur effectively protects the endothelium from oxidative stress‐induced injuries and inhibits the phenotypic transition of VSMCs from a contractile to a synthetic phenotype at inflammatory sites.^[^ ^10^ ^]^ These findings have led to the application of Cur in angioplasty, where it has been shown to promote reendothelialization and inhibit neointimal proliferation in previous studies.^[^ ^11^ ^]^ Nonetheless, the clinical utility of Cur is hindered by its poor water solubility and low bioavailability. Therefore, there is an urgent need to design biocompatible carriers capable of achieving a sustained release of Cur at the site of the injured artery. In addition, more efforts are required to fully elucidate the therapeutic effects of Cur in arterial remodeling.

Nitric oxide (NO), also known as endothelium‐derived relaxing factor, has been extensively studied for its role in vasodilation.^[^ ^12^ ^]^ NO primarily exerts its vasodilatory effects through the activation of guanylate cyclase, which converts guanosine triphosphate to cyclic guanosine monophosphate (cGMP). This subsequently stimulates cGMP‐dependent protein kinase, leading to the relaxation of VSMCs.^[^ ^13^ ^]^ Although the artery endothelium can produce endogenous NO to maintain vasodilation, the insufficient amount of endogenous NO resulting from endothelial denudation after angioplasty is unable to counteract constrictive remodeling effectively. Therefore, the application of exogenous NO donor has been exploited for their potential in anti‐restenosis strategies.^[^ ^14^ ^]^ However, accurately controlling the amount of NO released from NO donor is challenging, as excessive NO can lead to the generation of cytotoxic oxygen intermediates, while simultaneously shortening the effective treatment cycle of NO. Therefore, further endeavors are required for the on‐demand liberation of NO, particularly considering concentration‐dependent double‐edged role of NO.

**Figure 1:**
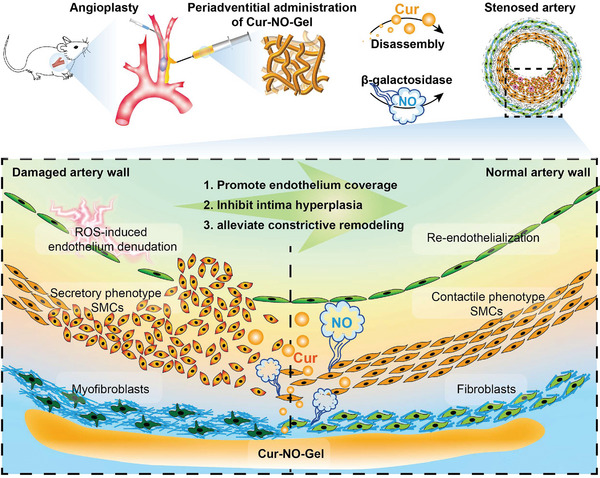
Schematic description of Cur‐NO‐Gel used in this study. Cur‐NO‐Gel, administered periadventitially, possesses the unique ability for sustained Cur release and on‐demand liberation of NO. This integrated hydrogel employs a three‐pronged approach to combat stenosis post‐angioplasty.

To address the aforementioned challenges, we developed a stent‐free hydrogel with the capability for sustained Cur release and on‐demand NO liberation. The integrated hydrogel exerted the therapeutic effect on vascular stenosis by three approaches: 1) promoting reendothelialization, 2) suppressing neointima hyperplasia, and 3) improving constrictive remodeling. The hydrogel used in our design is a self‐assembling peptide hydrogel (SAPH), which is a safe and biocompatible material used as a drug carrier for tissue repair and regeneration.^[^ ^15^ ^]^ By harnessing the π–π interactions of aromatic amino acids and leveraging the amphiphilicity of the hydrogelator architecture, we conjugated a hydrophilic sugar‐caged NO donor with an aromatic peptide of Nap‐FFGGG to create a hydrogelator capable of forming NO‐releasing SAPH (denoted as NO‐Gel). NO‐Gel can release NO by removing protective galactose from the NO donor under the catalysis of β‐galactosidase. Additionally, we encapsulated Cur into NO‐Gel to form the integrated hydrogel (denoted as Cur‐NO‐Gel). As illustrated in **Figure** **1**, when Cur‐NO‐Gel was periadventitially administered at the balloon injury site, it underwent gradual hydrolysis, resulting in the release of Cur. The released Cur played a protective role in vascular endothelial cells (VECs), shielding them from damage caused by reactive oxygen species (ROS). Moreover, it inhibited the detrimental phenotype transition of vascular smooth muscle cells (VSMCs) and adventitial fibroblasts. Simultaneously, the on‐demand release of NO triggered by β‐galactosidase could avoid the toxic side effects associated with unstable spontaneous release, exhibiting a synergistic vasodilatory effect with Cur, thereby compensating for the limitations of Cur in the treatment of constrictive remodeling. Consequently, Cur‐NO‐Gel exhibits the potential to efficiently improve vascular remodeling and combat restenosis after angioplasty.

## 2 Results

### 2.1 Preparation and Characterization of Cur‐NO‐Gel

**Figure 2:**
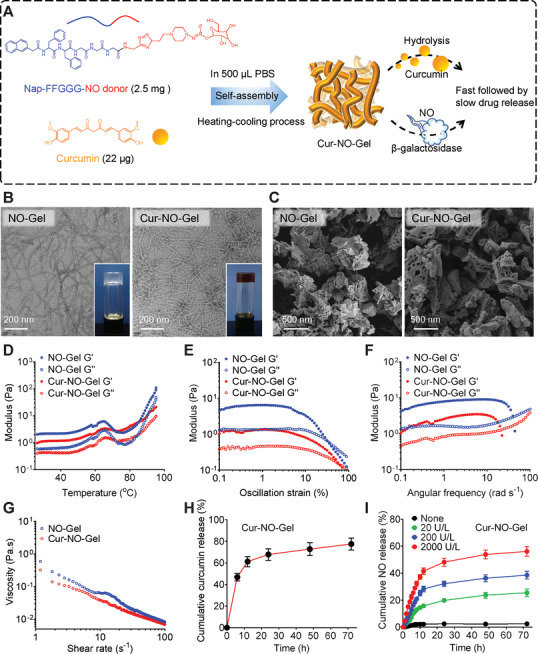
Preparation and characterization of Cur‐NO‐Gel. A) Illustration of preparation process and drug release for Cur‐NO‐Gel. B) TEM images of NO‐Gel and Cur‐NO‐Gel (inset: optical photographs of the formed gels). C) SEM images of NO‐Gel and Cur‐NO‐Gel. D) Evolution of storage modulus (G′) and loss modulus(G″) under different temperature (oscillation strain (γ) = 1%, angular frequency (ε) = 1 rad s^−1^, heating rate = 5 °C min^−1^). E) Evolution of G′ and G″ in the strain sweep measurement (ε = 1 rad s^−1^). F) Evolution of G′ and G″ in the frequency sweep test (γ = 1%). G) Viscosity of hydrogel versus shear rate. H) The accumulative release of Cur from Cur‐NO‐Gel at 37 °C. I) The release profile of NO from Cur‐NO‐Gel with the addition of different concentration of β‐galactosidase at 37 °C. Data are shown as mean ± SD.

To manipulate a three‐pronged approach to overcome the stenosis after angioplasty, we prepared Cur‐NO‐Gel, which was formed by assembling a conjugate of a NO donor‐peptide as a hydrogelator and encapsulating payloads of Cur (**Figure** **2**A). The synthesis of the hydrogelator (Nap‐FFGGG‐NO donor) is outlined in Figure S1 (Supporting Information). The molecular structure of the hydrogelator was confirmed using high‐resolution mass spectrometry (HR‐MS) and ^1^H nuclear magnetic resonance (^1^H NMR) (Figure S2A,B, Supporting Information). Cur‐NO‐Gel was prepared through a heating‐cooling process of the hydrogelator solution, followed by adding Cur solution. As a control, we also prepared NO‐Gel without Cur under identical conditions. The hydrogels (inset images on the bottom right corner of Figure 2B) remained stable for at least one month. The microstructures of the hydrogels were observed using transmission electron microscopy (TEM). NO‐Gel consisted of entangled long nanofibers with diameters of 10.40 ± 1.59 nm, while Cur‐NO‐Gel exhibited relatively coarse nanofibers with diameters of 16.06 ± 1.40 nm (Figure 2B). The significant morphological change indicated successful incorporation of Cur into the nanofibers. Scanning electron microscopy (SEM) further illustrated the microstructures of NO‐Gel and Cur‐NO‐Gel, and energy‐dispersive X‐ray spectroscopy mapping validated the presence of nitrogen elements in the nanofibers (Figure 2C; Figure S2C,D, Supporting Information).

The dynamic rheological properties of both NO‐Gel and Cur‐NO‐Gel were characterized to investigate the gelation performance of these hydrogels. Initially, we measured storage modulus (G′) and loss modulus(G″) of the hydrogelator solutions on a rheometer under a temperature scan mode. As the temperature decreased, both G′ and G″ of the hydrogelator solutions decreased simultaneously. In addition, the difference between the two moduli gradually widened, indicating a potential transition of the hydrogelator solutions from a liquid state to a gel state within the temperature range of 25 to 95 °C (Figure 2D). The dynamic strain‐sweep results revealed a wide linear viscoelastic range in the hydrogels at low strain (Figure 2E). At higher strain, both G′ and G″ decreased concurrently, suggesting a shear‐thinning behavior in the hydrogels. Frequency sweep tests performed at 1% strain exhibited slight increments in both G′ and G″ of the hydrogels with rising frequency. However, at 10 rad s^−1^, a sudden decline in G′ was observed, demonstrating favorable frequency‐dependent viscoelastic behavior (Figure 2F). Additionally, their viscosity notably decreased with increasing shear rate (Figure 2G). The shear‐thinning property is favorable for periadventitial injection and is also consistent with the modulus results mentioned above. Considering the intended periadventitial application of Cur‐NO‐Gel, we tracked the G′ and G″ of Cur‐NO‐Gel at various hydrolysis stages (Figure S2E, Supporting Information). Cur‐NO‐Gel exhibited a G′ exceeding its G″, signifying its continual maintenance of a gel state. Simultaneously, the gradual reduction in the discrepancy between these moduli indicated the progressive degradation of Cur‐NO‐Gel. We also monitored the release profiles of NO and Cur from Cur‐NO‐Gel. The release of Cur exhibited a rapid initial phase, with 61.22 ± 3.17% released within the first 12 h, followed by a slower release phase, resulting in a total release of 77.56 ± 3.78% within 72 h (Figure 2H). Similarly, the release of NO from Cur‐NO‐Gel also exhibited a “fast followed by slow” profile (Figure 2I). In addition, the liberated NO could be controlled by varying the concentration of β‐galactosidase added to the release medium. Given the sustained‐release behavior of both Cur and NO, Cur‐NO‐Gel shows promise as a potential drug in angioplasty.

### 2.2 Cur‐NO‐Gel Preserves VECs Against Oxidative Stress Injury

**Figure 3:**
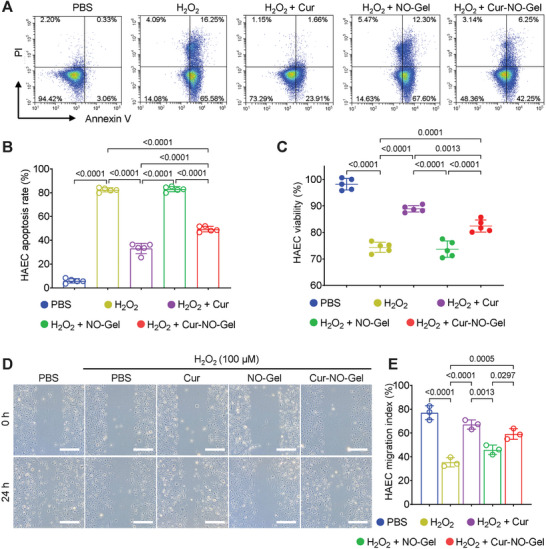
Cur‐NO‐Gel preserves VECs against oxidative stress injury. A) HAEC apoptosis after treated with different compounds measured by flow cytometry (Cur concentration, 25 µM; NO donor concentration, 75 µM) for 24 h. B) Quantitation of apoptosis rate of HAECs in different groups (n = 5). C) The viability of HAECs after the indicated treatments was quantitatively analyzed (n = 5). D) Scratch‐wound assay of HAECs. Scale bar: 500 µm. E) Quantitation of HAEC migration index in different groups (n = 3). β‐galactosidase was added to all culture media containing hydrogel at a dose of 0.2 U mL^−1^. Data are shown as mean ± SD.

Mechanical damage caused by angioplasty can arouse severe oxidative stress, leading to impaired reendothelialization process.^[^ ^16^ ^]^ To simulate this process in vitro, we incubated human aortic endothelial cells (HAECs) with various concentrations of H_2_O_2_ for 24 h and assessed cell viability (Figure S3, Supporting Information). The results indicated that a concentration of 100 µM H_2_O_2_ was suitable for inducing oxidative stress damage to HAECs, and hence this concentration was chosen for subsequent experiments. First, we examined the anti‐apoptosis effect of Cur‐NO‐Gel on HAECs (**Figure** **3**A,B). In the H_2_O_2_ group, 82.38 ± 1.62% of cells underwent apoptosis, while the ratio of the apoptotic cells significantly decreased to only 33.11 ± 3.96% in the Cur group. Cur‐NO‐Gel also reduced the percentage of apoptotic cells (49.40 ± 2.11%), whereas NO‐Gel had a limited impact on the apoptosis rate (83.03 ± 1.90%). These findings suggest that the anti‐apoptotic effect of Cur‐NO‐Gel is mainly attributed to the sustained release of Cur. Cell counting kit‐8 (CCK‐8) assay further demonstrated that Cur‐NO‐Gel preserved the viability of HAECs treated with H_2_O_2_, which was in agreement with the results of apoptosis analysis (Figure 3C). Additionally, we evaluated the migration capacity of HAECs using the migration index (Figure 3D,E). Treatment with H_2_O_2_ led to a significant decrease in the migration index from 77.04 ± 4.09% to 35.38 ± 2.89%, which was restored to 67.15 ± 2.69% by Cur. Meanwhile, Cur‐NO‐Gel could enhance the migration index to 59.20 ± 3.12%. These lines of evidences indicate that Cur‐NO‐Gel has the potential to protect VECs against oxidative stress injury.

### 2.3 Cur‐NO‐Gel Inhibits The Activity of VSMCs and Promotes Contractile‐Phenotype Transition

**Figure 4:**
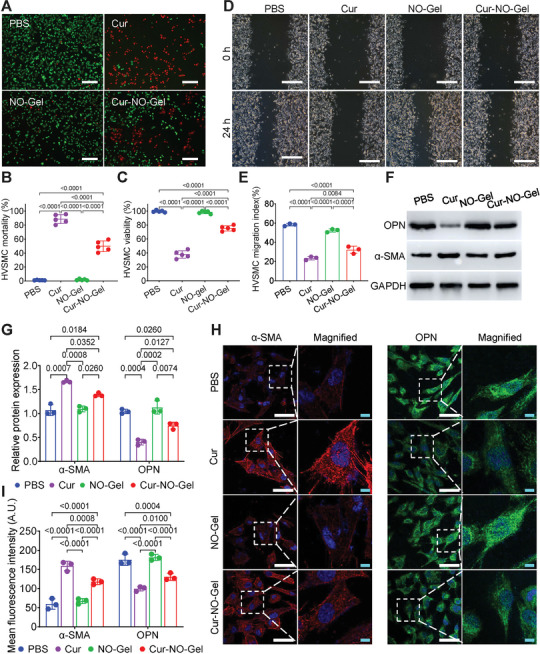
Cur‐NO‐Gel inhibits the activity of VSMCs and promotes contractile‐phenotype transition. A) Representative images of Calcein‐AM/PI Double Stain assay showing HVSMCs treated with different compounds (Cur concentration, 25 µM; NO donor concentration, 75 µM) for 24 h. Scale bar: 200 µm. B) Quantitation of HVSMC mortality (n = 5). C) Quantitative analysis of HVSMC viability in different groups (n = 5). D) Scratch‐wound assay of HVSMCs. Scale bar: 500 µm. E) Quantitation of VSMC migration index in different groups (n = 3). F) and G) Western blot analysis of α‐SMA and OPN expression of HVSMCs. H) and I) Immunofluorescence staining exhibiting α‐SMA and OPN expression in HVSMCs (n = 3). β‐galactosidase was added to all culture media containing hydrogel at a dose of 0.2 U mL^−1^. Scale bar: 50 µm (white) and 5 µm (blue). Data are shown as mean ± SD.

VSMC activation and neointima hyperplasia are the main causes of vascular stenosis after angioplasty. In this work, we explored the inhibiting effect of Cur‐NO‐Gel on HVSMC activity. To assess the toxicity of Cur‐NO‐Gel on HVSMCs, we performed Live/dead cell double stain. As shown in **Figure** **4**A,B, compared with the PBS group, we observed a reduction in green fluorescence signals (indicating live cells) and an increase in red fluorescence signals (indicating dead cells) in the Cur group and the Cur‐NO‐Gel group, highlighting the potent cytotoxicity of Cur‐NO‐Gel against VSMCs. In addition, we found that Cur‐NO‐Gel significantly reduced HVSMC viability by up to 24.69 ± 0.76% (Figure 4C). The migration ability of HVSMCs in different groups was also evaluated. As depicted in Figure 4D,E, the migration index of HVSMCs decreased from 58.25 ± 1.35% in the PBS group to 32.13 ± 3.75% in the Cur‐NO‐Gel group, indicating the strong inhibitory effect of Cur‐NO‐Gel on VSMC migration.

Under normal conditions, VSMCs are usually in a quiescent state with a low turnover rate. However, after angioplasty, VSMCs undergo a phenotype transition from a contractile to a synthetic phenotype, resulting in a down‐regulation of alpha‐smooth muscle actin (α‐SMA) and an up‐regulation of osteopontin (OPN).^[^ ^17^ ^]^ Therefore, we analyzed the phenotype alteration of HVSMCs treated with various compounds. Western blot analysis revealed that HVSMCs treated with Cur‐NO‐Gel displayed elevated expression of α‐SMA and decreased expression of OPN (Figure 4F,G). Furthermore, we conducted immunofluorescence staining for α‐SMA (red fluorescence signals) and OPN (green fluorescence signals) (Figure 4H,I). Compared with the PBS group, the Cur‐NO‐Gel group displayed stronger signals of α‐SMA and weaker signals of OPN. Together, these findings indicate that Cur‐NO‐Gel can promote the transition to a contractile phenotype of VSMCs, which may be associated with the suppression of VSMC activities.

### 2.4 Cur‐NO‐Gel Suppresses The Activity of Vascular Fibroblasts and Inhibits Myofibroblast Transformation

**Figure 5:**
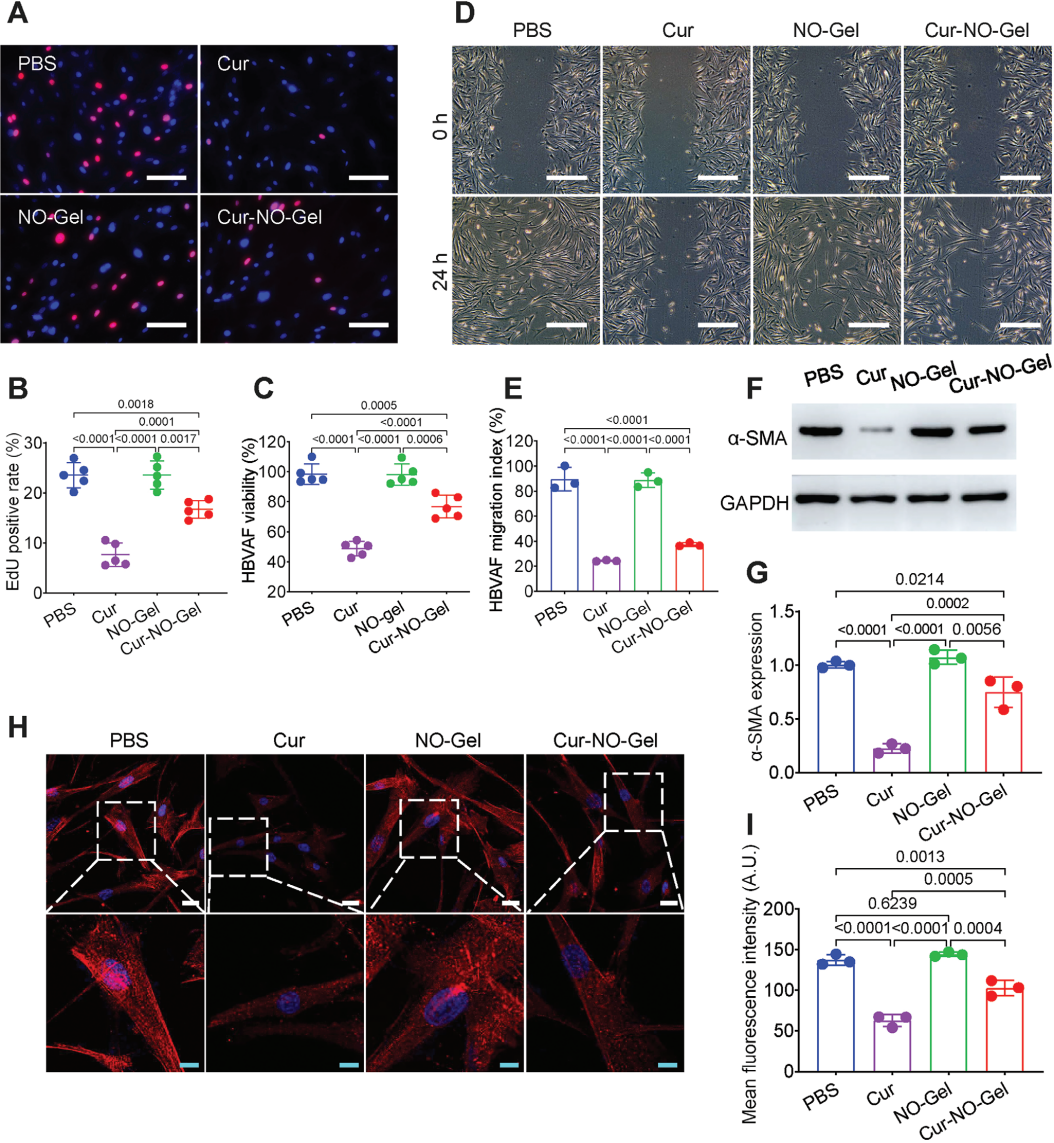
Cur‐NO‐Gel suppresses the activity of vascular fibroblasts and inhibits myofibroblast transformation. A) Representative photographs of EDU staining of HBVAFs treated with different compounds (Cur concentration, 25 µM; NO donor concentration, 75 µM) for 24 h. Scale bar: 200 µm. B) Quantitation of EdU‐positive rate of HBVAFs in different groups (n = 5). C) Quantitative analysis of HBVAF viability in different groups (n = 5). D) Scratch‐wound assay of HVSMCs. Scale bar: 500 µm. E) Quantitation of HBVAF migration index in different groups (n = 3). F) and G) Western blot analysis of α‐SMA expression of HBVAFs in different groups. H) and I) Immunofluorescence staining of HBVAFs for α‐SMA expression (n = 3). β‐galactosidase was added to all culture media containing hydrogel at a dose of 0.2 U mL^−1^. Scale bar: 50 µm (white) and 5 µm (blue). Data are shown as mean ± SD.

The role of arterial adventitia in artery remodeling after angioplasty has historically been overlooked in comparison to the intima and media layers. However, recent research has unequivocally revealed that adventitial remodeling, mediated by hyper‐activated fibroblasts, plays a crucial part in constrictive remodeling after angioplasty.^[^ ^18^ ^]^ To investigate the potential inhibitory effect of Cur‐NO‐Gel on constrictive remodeling, we conducted an evaluation of its effect on human brain vascular adventitial fibroblasts (HBVAFs). We performed a proliferating cell staining (EdU assay) on HBVAFs treated with different compounds (**Figure** **5**A) and quantified the proportion of proliferating cells (red fluorescence signals) (Figure 5B). The results showed that Cur could restrain the proliferation rate from 23.52 ± 0.50% to 7.63 ± 0.47%, while Cur‐NO‐Gel lowered the proliferation rate to 16.7 ± 0.35%. The findings obtained from the CCK‐8 cell viability assay were consistent with those from the EdU assay (Figure 5C). It has been observed that activated fibroblasts have the ability to migrate towards neointima and accelerate stenosis of the injured artery.^[^ ^19^ ^]^ Therefore, we assessed the repression of Cur‐NO‐Gel on the migration ability of HBVAFs using a scratch‐wound assay. The results revealed that Cur‐NO‐Gel reduced the migration index from 89.64 ± 9.33% (the PBS group) to 37.23 ± 1.49% (Figure 5D,E). These results imply that Cur‐NO‐Gel can effectively inhibit the activity of vascular fibroblasts.

Activated fibroblasts, also known as myofibroblasts, are characterized by their high expression of α‐SMA.^[^ ^20^ ^]^ Therefore, we investigated the α‐SMA expression in HBVAFs treated with Cur‐NO‐Gel. As presented in Figure 5F,G, the protein level of α‐SMA was significantly decreased in HBVAFs treated with Cur‐NO‐Gel compared with the PBS group. In addition, we evaluated α‐SMA expression using immunofluorescence staining (Figure 5H,I). The strong red fluorescence signals in the PBS group and weak signals in the Cur‐NO‐Gel group indicated that Cur‐NO‐Gel could inhibit the expression of α‐SMA in HBVAFs, which is consistent with the results of western blot analysis. These results prove that Cur‐NO‐Gel can inhibit activation of adventitial fibroblasts into myofibroblasts.

### 2.5 Cur‐NO‐Gel Prevents Vascular Stenosis After Carotid Balloon Angioplasty

**Figure 6:**
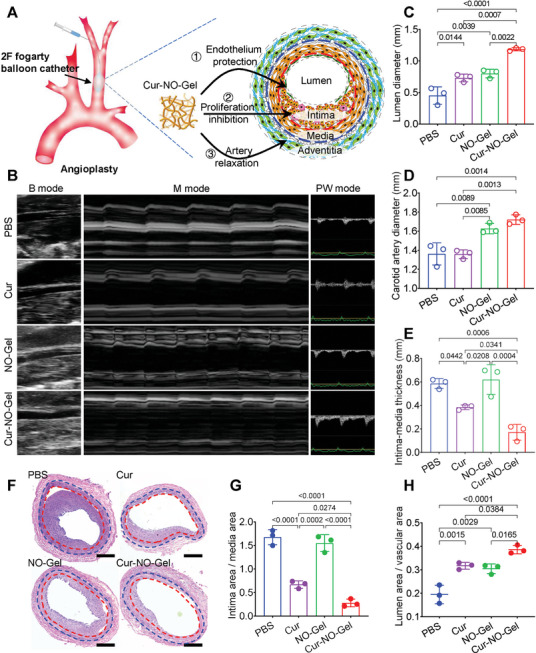
Cur‐NO‐Gel prevents vascular stenosis after carotid balloon angioplasty. A) Illustration of the construction of balloon angioplasty model and Cur‐NO‐Gel treatment for vascular remodeling. B) Representative USI images of different groups two weeks after angioplasty. C) and D) Quantitative analysis of the lumen diameter and vascular diameter of the injured arteries (n = 3). E) Quantitation of intima‐media thickness, including neointima layer and media layer of artery. F) Representative sections from injured arteries in different groups. Scale bar: 500 µm. G) and H) Quantified data of intimal hyperplasia (ratio of intima/media area) and lumen area (ratio of lumen/vascular area). All the rats in the hydrogel groups were injected with β‐galactosidase (300 µL, 0.5 mg mL^−1^) via tail vein every day for 7 consecutive days since angioplasty. Data are shown as mean ± SD.

After demonstrating the regulation effect of Cur‐NO‐Gel on vascular cells in vitro, we investigated its therapeutic effect on vascular remodeling in a rat balloon angioplasty model (**Figure** **6**A; Figure S4A, Supporting Information). Cur‐NO‐Gel was applied to the outside of the injured segment of the carotid artery, as periadventitial administration has been reported as an effective approach for delivering various drugs, including small‐molecule drugs and nanoparticles, into the injured rat carotid arterial wall.^[^ ^21^ ^]^ Two weeks after the surgery, vascular remodeling and the therapeutic effect of Cur‐NO‐Gel were monitored by ultrasound imaging (USI). The injured carotid artery was scanned to estimate vascular remodeling‐related indicators, including carotid artery diameter, lumen diameter, intima‐media thickness, pulse delay time (PDT), and blood velocity (Figure 6B). M‐mode USI indicated that the values of lumen diameter were significantly higher in the Cur‐NO‐Gel group than in the other groups (Figure 6C). In addition, both the Cur‐NO‐Gel group and NO‐Gel group showed increased artery diameter compared with the PBS group (Figure 6D). We also observed a thinner intima‐media thickness at the injured artery in the Cur‐NO‐Gel group and Cur group compared with the other groups (Figure 6E). These results confirmed that Cur‐NO‐Gel could prevent vascular stenosis by inducing NO‐mediated vasodilation and Cur‐mediated neointima inhibition. PW‐mode analysis for the Cur‐NO‐Gel group yielded higher PDT values than those obtained for the PBS group, and blood velocity in the Cur‐NO‐Gel group was faster than that in the PBS group (Figure S4B,C, Supporting Information). These indicators demonstrate that Cur‐NO‐Gel can induce beneficial vascular remodeling and improve artery function.

The sections of injured arteries were collected and stained with hematoxylin‐esosin (H&E) and Masson staining for morphometric analysis (Figure 6F; Figure S4D, Supporting Information). The intima/media area ratio decreased from 1.67 (PBS group) to 0.67 (Cur group) and to 0.31 (Cur‐NO‐Gel group) (Figure 6G). Additionally, the ratio of lumen area to vascular area increased by 1.7‐fold in the Cur group, 1.6‐fold in the NO‐Gel group, and 2.0‐fold in the Cur‐NO‐Gel group compared with the PBS group (Figure 6H). Consistent with the analysis from USI, these results demonstrate that Cur‐NO‐Gel can effectively inhibit neointima formation and preserve lumen area, thereby improving artery remodeling and preventing stenosis after angioplasty.

### 2.6 Cur‐NO‐Gel Plays A Three‐Pronged Role In Anti‐Stenosis After Angioplasty

To further elucidate the therapeutic effect of Cur‐NO‐Gel on artery remodeling after angioplasty, we investigated its efficacy in addressing three crucial pathological processes: delayed reendothelialization, neointima hyperplasia, and constrictive remodeling.

**Figure 7:**
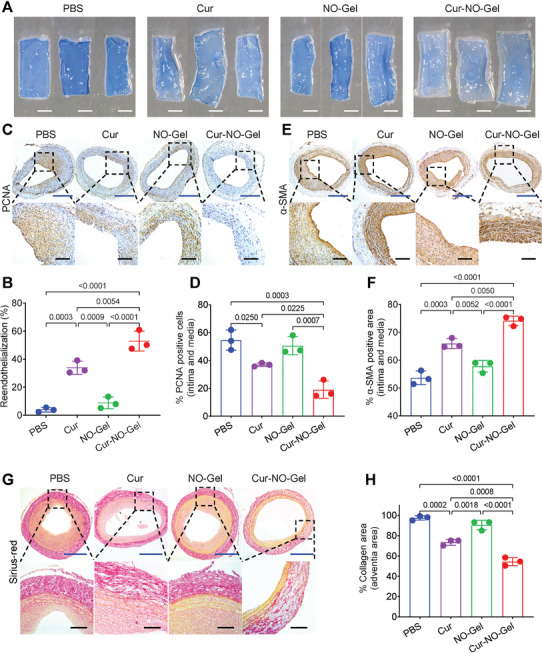
Cur‐NO‐Gel plays a three‐pronged role in preventing stenosis after angioplasty. A) Evans Blue staining of common carotid artery harvested from rats after treatment (NO donor, 0.15 µmol per rat; Cur dosage, 13 µg per rat). Scale bar: 2 mm. B) Quantitation of accumulation of Evans Blue in different groups (n = 3). C) Representative microscopic images of PCNA‐stained sections of carotid arteries. Scale bar: 500 µm (blue) and 100 µm (black). D) Quantified data of PCNA positive cells (intima and media) (n = 3). E) α‐SMA immunostaining sections of carotid arteries in different groups. Scale bar: 500 µm (blue) and 100 µm (black). F) Quantitation of α‐SMA positive area (intima and media) (n = 3). G) Sirius Red staining sections of carotid arteries. Scale bar: 500 µm (blue) and 100 µm (black). H) Quantitative analysis of collagen content in adventitia area (n = 3). Data are shown as mean ± SD.

To evaluate reendothelialization in the injured vessels, Evans Blue staining was performed as shown in **Figure** **7**A. The area without endothelial coverage was stained blue, while the area with reendothelialization remained their original white color. This distinct contrast in staining allowed us to easily differentiate between regions with vascular damage and those currently undergoing reendothelialization. Quantitative results of reendothelialization in Figure 7B indicated the percentages of reendothelialization area were 3.87 ± 1.32% (PBS group), 33.87 ± 3.76% (Cur group), 8.78 ± 3.56% (NO‐Gel group), and 52.85 ± 5.79% (Cur‐NO‐Gel group), respectively. Additionally, CD31 (a VEC marker) staining showed that the reendothelialization rate in the Cur‐NO‐Gel group was 2.1‐fold higher compared with the PBS group, corroborating the findings of Evans Blue staining (Figure S5A,B, Supporting Information). These observations illustrate that Cur‐NO‐Gel can promote reendothelialization after angioplasty.

Neointima hyperplasia was assessed by Proliferating Cell Nuclear Antigen (PCNA) staining (Figure 7C,D). The PBS group exhibited the largest PCNA positive area (20.81 ± 3.05%), which significantly decreased to 5.00 ± 1.40% in the Cur‐NO‐Gel group, indicating effective inhibition of neointima hyperplasia. Immunohistochemical staining was conducted to characterize the phenotypic switch markers of VSMCs. The quantification of immunohistochemical analysis demonstrated that Cur‐NO‐Gel upregulated the expression of α‐SMA (a contractile marker) (Figure 7E,F) and decreased the expression of OPN (a synthetic phenotype maker) (Figure S5C,D, Supporting Information). The phenotypic switching of VSMCs stimulated by Cur‐NO‐Gel provides evidence of its ability to inhibit neointima hyperplasia.

Adventitial collagen deposition has been considered a critical contributor to constrictive remodeling.^[^ ^22^ ^]^ In this study, we evaluated the accumulation of collagen in adventitia using Sirius Red staining (Figure 7G,H). Compared with the PBS group, the collagen‐laden area in the Cur‐NO‐Gel group decreased from 97.57 ± 2.16% to 54.58 ± 4.03%, while the area in the Cur group was only reduced to 73.57 ± 2.88%. These results, combined with the USI analysis, indicate the potential of Cur‐NO‐Gel to effectively alleviate constrictive remodeling by replenishing NO production and reducing the secretion of adventitial collagen.

To explore the biocompatibility of Cur‐NO‐Gel, we collected major organs from each group of rats post‐euthanasia for H&E staining, revealing no significant hydropic damage or necrotic lesions (Figure S6, Supporting Information).

In summary, Cur‐NO‐Gel can play a favorable role in promoting reendothelialization, suppressing neointima hyperplasia, and improving constrictive remodeling, ultimately inhibiting artery stenosis after angioplasty.

## 3 Discussion

Although some drug‐eluting stents have been reported, most drug coating materials focus solely on inhibiting neointima hyperplasia or promoting reendothelialization, while often overlooking the role of constrictive remodeling in vascular restenosis. It is known that in balloon‐injured arteries, activated adventitial fibroblasts transform into myofibroblasts, followed by proliferation and excessive collagen secretion, leading to adventitial thickening and constrictive remodeling.^[^ ^23^ ^]^ Therefore, developing an integrated anti‐restenosis strategy is urgently needed to improve patients’ prognosis after angioplasty. We included a table that compares the performance differences between our integrated arterial remodeling hydrogel and related drug‐eluting stents in preventing stenosis after angioplasty (Table S1, Supporting Information).

Previous studies have indicated that NO hydrogels can effectively promote endothelial restoration, inhibit neointimal proliferation, and suppress thrombus formation post‐angioplasty.^[^ ^24^ ^]^ In this research, an NO hydrogel was combined with Cur and applied to prevent restenosis following angioplasty. We have developed an integrated Cur‐NO‐Gel, which allows for controlled and gradual drug release at the specific site of arterial injury. This innovative gel formulation consists of a NO donor‐peptide conjugate as a hydrogelator, wherein Cur is encapsulated during the assembly process. Upon periadventitial administration at the balloon injury site, Cur‐NO‐Gel underwent gradual hydrolysis, releasing Cur. After intravenous administration of β‐galactosidase, the hydrogel also liberated NO. Although there is an initial burst release of Cur within the first 12 h, this early release phase may be beneficial for inhibiting acute inflammation in the damaged artery. After the first 12 h, Cur‐NO‐Gel exhibited a nearly linear sustained‐release profile, with Cur being released at an average rate of 0.27% per hour. In addition, the release of NO could be fully controlled by β‐galactosidase, enabling on‐demand NO delivery and extended periods of effective NO treatment.

Oxidative damage to VECs and excessive activation of VSMCs/fibroblasts are key factors leading to detrimental vascular remodeling. Here, we included a table that comprehensively summarizes and quantifies the effects and interventions of the compounds in this study both in vitro and in vivo (Table S2, Supporting Information). The limited impact of NO‐Gel on vascular cell activity suggests that relying solely on NO may not be sufficient to improve vascular remodeling. In addition, the notable effect of free Cur on vascular cells in vitro compared to Cur‐NO‐Gel might be attributed to the slow‐release characteristic of Cur in SAPH. USI showed that Cur‐NO‐Gel could visibly dilate the lumen and artery diameter of the injured artery through NO‐induced vasodilation and Cur‐mediated neointima inhibition, which agreed with those arising from H&E staining.

To further elucidate the mechanism responsible for the therapeutic effect of Cur‐NO‐Gel on artery remodeling after angioplasty, we investigated its efficacy on three crucial pathological processes: delayed reendothelialization, neointima hyperplasia, and constrictive remodeling. Evans Blue assay and CD31 staining analysis showed that Cur‐NO‐Gel promoted reendothelialization after angioplasty. PCNA staining indicated the inhibitory effect of Cur‐NO‐Gel on VSMC proliferation, thereby attenuating neointima hyperplasia. Immunohistochemical analysis confirmed that Cur‐NO‐Gel promoted the transition of smooth muscle cell into a contraction phenotype. Furthermore, Sirius Red staining showed that Cur‐NO‐Gel significantly reduced the deposition of collagen in the adventitia, thereby dilating the blood vessels.

Combing the pharmacological characteristics of Cur and the biological function of NO, the periadventitial administration of Cur‐NO‐Gel has demonstrated its ability to effectively inhibit vascular stenosis after angioplasty by targeting the three main pathological processes involved in vascular remodeling. Furthermore, it is worth noting that SAPH holds great potential as an ideal drug coating material for tissue regeneration scaffolds. In this context, the utilization of Cur‐NO‐Gel in drug‐eluting stents for the prevention of stenosis after angioplasty becomes an intriguing prospect. However, it is important to acknowledge that our current investigation primarily focused on evaluating the therapeutic effect of Cur‐NO‐Gel in treating artery stenosis, neglecting the exploration of its potential application as a drug coating on balloons or stents. Therefore, conducting further investigation is warranted to assess the efficacy of utilizing Cur‐NO‐Gel as a coating for stents or balloon catheters. By doing so, the newly designed SAPH could potentially serve as a valuable theoretical reference for the development of a new generation of drug coatings with potential clinical applications in the field of angioplasty.

## 4 Experimental Section

### Materials

Fmoc‐amino acids and solvents for solid phase synthesis of peptide derivatives were bought from Bidepharm (Shanghai, China) and Macklin (Shanghai, China), respectively. Cur was purchased from Energy Chemical (Beijing, China). O‐(benzotriazole‐1‐yl)‐N,N,N′,N′‐tetramethyluroniumhexafluorophosphate (HBTU), N,N‐Diisopropylethylamine (DIEA), 2‐(naphthalen‐2‐yl) acetic acid, triisopropylsilane, and trifluoroacetic acid (TFA) were obtained from Innochem (Beijing, China). All other chemicals were reagent grade or better and used without further purification.

### Preparation and Characterization of Hydrogel

The NO donor was provided by Yang's team from Nankai University. The Nap‐FFGGG‐NO donor peptide derivative (NO‐PEP) was prepared as previously reported.^[^ ^25^ ^]^ To prepare Cur‐NO‐Gel, 5 mg of Cur was dissolved in 5 mL of DMSO and stirred at 100 °C. At the same time, 2.5 mg of NO‐PEP dissolved in 500 µL of PBS (pH 7.4) was heated to boiling point. Then, 22 µL of Cur solution at 100 °C was added to the NO‐PEP solution. The hot mixture was cooled to ambient temperature, and the Cur‐NO‐Gel formed within 5 minutes.

The compounds prepared in this study were purified by high performance liquid chromatography (HPLC, Agilent, California, America) using C18 RP column. ^1^H NMR spectroscopy was recorded on a Bruker AVANCE III400 spectrometer. HR‐MS spectra were obtained using an Orbitrap Fusion Tribrid mass spectrometer (Thermo Fisher Scientific, Massachusetts, America). The mechanical strength of the hydrogel was evaluated using a rheometer (DHR‐2, Waters, Massachusetts, America). TEM images were captured using a FEI Tecnai G2 Spirit transmission electron microscope (Thermo Fisher Scientific, Massachusetts, America). SEM images were acquired using a scanning electron microscope (ZEISS GeminiSEM 300, Germany).

For assessing the controllable NO release, 500 µL of PBS containing β‐galactosidase at various concentrations (0, 20, 200, and 2000 U L^−1^) was placed atop 500 µL of hydrogel at 37 °C. Liberated NO in the upper solution was detected using a micro NO content assay kit (Beyotime Biotech, Shanghai, China). The Cur release was evaluated using a similar approach. A microplate reader (Bio‐RAD iMark™, California, America) was used to detect the optical density at 420 nm and calculated the cumulative Cur release using standard absorbance intensity measurement and curve fitting methods.

### Cell Culture and Viability Assay

Human aortic endothelial cells (HAECs), human vascular smooth muscle cells (HVSMCs), and human brain vascular adventitial fibroblasts (HBVAFs) were bought from ATCC (Virginia, America). HAECs were cultured in endothelial cell medium, while HVSMCs and HBVAFs were cultured in DMEM and MEM medium, respectively, supplemented with 10% FBS and 1% penicillin‐streptomycin. All cell culture media and reagents were purchased from Gibco (California, America).

The viability of HAECs was assessed using a cell counting kit‐8 (CCK‐8) (KeyGEN Biotechnology, Jiangsu, China). After 24 h of attachment, HAECs were divided into five groups: PBS, H_2_O_2_ (100 µM), H_2_O_2_ + Cur (25 µM), H_2_O_2_ + NO‐Gel (NO donor, 75 µM), and H_2_O_2_ + Cur‐NO‐Gel (Cur concentration, 25 µM), and incubated for 24 h. β‐galactosidase was added to all culture media containing NO donor at a dose of 0.2 U mL^−1^. Then, the cells were incubated with CCK‐8 assay buffer and detected using a microplate reader (Bio‐RAD iMark™, California, America). The viability of HVSMCs and HBVAFs was evaluated using a similar approach.

### Scratch‐Wound Assay

Cells in the exponential growth phase were seeded in 6‐well plates and allowed to grow to confluence. Subsequently, a sterile pipette tip was used to gently create a wound in the center of each well. The cells were then treated in the same manner as in the cell viability assay. Microscopic images of the region marked along the wound area were captured, and the wounded area was calculated using ImageJ software (version 1.46r). The migration index of cells was estimated using the following formula:

(1) $$\mathrm{Migration}\;\mathrm{index}\;=\frac{\left(\mathrm{Wounded}\;\mathrm{area}\;\mathrm{at}\;0\;h\right)-(\mathrm{Wounded}\;\mathrm{area}\;\mathrm{at}\;24\;h)}{\mathrm{Wounded}\;\mathrm{area}\;\mathrm{at}\;0\;h}$$

### Apoptosis Assay

Apoptosis assay was conducted using Annexin V‐FITC/PI Apoptosis Detection Kit (KeyGEN Biotechnology, Jiangsu, China). After washing with PBS, HAECs were incubated with Annexin V‐EGFP and Propidium Iodide. Finally, the apoptotic cells were detected using a Beckman Coulter flow cytometer.

### Calcein‐AM/PI Double Stain Assay

Living cells and dead cells were detected using Calcein‐AM/PI double stain kit (Solarbio, Beijing, China). Once attached to the plate, HVSMCs were incubated in the culture medium containing Cur (25 µM), NO‐Gel (NO donor, 75µM ), or Cur‐NO‐Gel (Cur concentration, 25 µM) for 24 h, respectively. β‐galactosidase was added to all culture media containing NO donor at a dose of 0.2 U mL^−1^. Then, the cells were incubated with assay buffer according to the manufacturer's instruction and detected using a laser scanning confocal microscope (Leica TCS SP8, Wetzlar, Germany) with 490 nm and 545 nm excitation. Cells displaying green fluorescence were identified as viable/live cells, whereas those exhibiting red fluorescence were classified as non‐viable/dead cells.

### EdU Assay

After cell attachment, the cells were incubated in the culture medium supplemented with Cur (25 µM), NO‐Gel (NO donor, 75µM ), or Cur‐NO‐Gel (Cur concentration, 25 µM) for 24 hours. β‐galactosidase was added to all culture media containing the NO donor at a concentration of 0.2 U mL^−1^. Subsequently, the cells were treated with EdU assay buffer and examined using a laser scanning confocal microscope (Leica TCS SP8, Wetzlar, Germany) with excitation at 594 nm. Proliferating cells were identified as those displaying red fluorescence.

### Immunofluorescence Analysis

After treatment with different compounds, the cells were stained overnight at 4 °C with either α‐SMA or osteopontin antibody (ab124964, ab8448, Abcam, Cambridge, UK). After rinsing with PBS, the cells were then incubated at room temperature for 2 hours with goat anti‐rabbit IgG labeled with Alexa Fluor 488 or Cy3. Subsequently, the cells were stained with DAPI solution and observed using a laser scanning confocal microscope (Leica TCS SP8, Wetzlar, Germany). The average fluorescence intensity was calculated using ImageJ software (version 1.46r).

### Balloon Angioplasty Model

Sprague‐Dawley rats (250‐300 g) were obtained from Laboratory Animal Center of Southern Medical University. Balloon angioplasty was conducted as previously reported.^[^ ^26^ ^]^ In brief, the rats were anesthetized by intraperitoneal injection of pentobarbital sodium (50 mg kg^−1^). A midline cervical incision was made to expose the left common carotid artery. Subsequently, a 2F Fogarty balloon catheter was carefully inserted into the artery through an arteriotomy in the external carotid artery. To induce injury, the balloon was inflated to 4 atmospheres and held for 3 minutes before being deflated to 0 atmospheres. This procedure was replicated three times, after which the balloon was gently withdrawn at 1 atmosphere. An equal volume (30 µL) of Cur‐NO‐Gel or other control compounds (Cur dosage, 13 ug per rat) were applied around the injured artery. Free Cur was dispersed in Cremophor/PBS (1:10). All the rats in the hydrogel groups were injected with β‐galactosidase (300 µL, 0.5 mg mL^−1^) via tail vein every day for 7 consecutive days after angioplasty. Two weeks after the operation, the injured arteries were harvested for further experiments. All experimental procedures were approved by Institutional Animal Care and Use Committee of The Second Affiliated Hospital of Guangzhou Medical University and conducted in accordance with their guidelines (Acceptance number: B2022‐051).

### Evans Blue Assay

Two weeks after balloon angioplasty, 0.5 mL of 2% Evans Blue Dye (Macklin, Shanghai, China) was injected intravenously. After 20 minutes, the rats were perfused with PBS and the injured arteries were obtained and longitudinally opened. Evans Blue can easily penetrate the arterial wall in areas where the endothelium was denuded, but it was difficult to stain areas where the endothelium was intact. The extent of staining by Evans Blue within the vascular wall was quantified using ImageJ software (version 1.46r).

### Immunohistochemical Staining of Carotid Arteries Sections

Immunohistochemical staining was conducted on sections of the carotid artery harvested two weeks after balloon angioplasty. Briefly, the sections were incubated with each primary antibody overnight at 4 °C as follow: anti‐CD31 rabbit pAb (1:100), anti‐PCNA rabbit pAb (1:300), anti‐alpha SMA rabbit pAb (1:500), and anti‐OPN rabbit pAb (1:500). Subsequently, the sections were incubated at room temperature for 2 hours with a HRP‐conjugated goat‐anti‐rabbit secondary antibody. The visualization of the sections was then achieved using 3,3‐diaminobenzidine (DAB). All the antibodies and reagents used in immunohistochemical staining were bought from Servicebio (Wuhan, China).

### Statistical Analysis

Continuous data were presented as mean ± standard deviation (SD), and were tested for normality using the D'Agostino Pearson or Shapiro‐Wilk test. Student's t test was performed to determine statistically significant differences between two groups. For multiple comparisons, data were analyzed using ANOVA followed by Tukey post hoc tests. The measurements were taken from distinct samples. Statistical analysis was conducted using GraphPad Prism 9 software.

## Conflict of Interest

The authors declare no conflict of interest.

## Supporting information

Supporting Information

## Data Availability

The data that support the findings of this study are available from the corresponding author upon reasonable request.
