# Supplementary material for: An Integrated Arterial Remodeling Hydrogel for Preventing Restenosis After Angioplasty
Source: Adv Sci (Weinh). 2024 Feb 11;11(15):2307063. doi: 10.1002/advs.202307063 (PMC11022711; doi:10.1002/advs.202307063)
Supplement: Supplementary file 1 — Supporting Information [file ADVS-11-2307063-s001.pdf]

ADVANCED SCIENCE
Open Access

Supporting Information

for *Adv. Sci.*, DOI 10.1002/advs.202307063

An Integrated Arterial Remodeling Hydrogel for Preventing Restenosis After Angioplasty

Chenxing Fu, Qiu Li, Minghui Li, Jiexin Zhang, Feiran Zhou, Zechuan Li, Dongyue He, Xinyi Hu, Xiaodong Ning, Wenjie Guo, Weirun Li, Jing Ma, Guoqin Chen, Yafang Xiao, Caiwen Ou* and Weisheng Guo**

Supporting Information

An Integrated Arterial Remodeling Hydrogel for Preventing Restenosis after Angioplasty

Chenxing Fu, Qiu Li, Minghui Li, Jiexin Zhang, Feiran Zhou, Zechuan Li, Dongyue He, Xinyi Hu, Xiaodong Ning, Wenjie Guo, Weirun Li, Jing Ma, Guoqin Chen, Yafang Xiao, Caiwen Ou*, and Weisheng Guo**

((Please insert your Supporting Information text/figures here. Please note: Supporting Display items, should be referred to as Figure S1, Equation S2, etc., in the main text…)

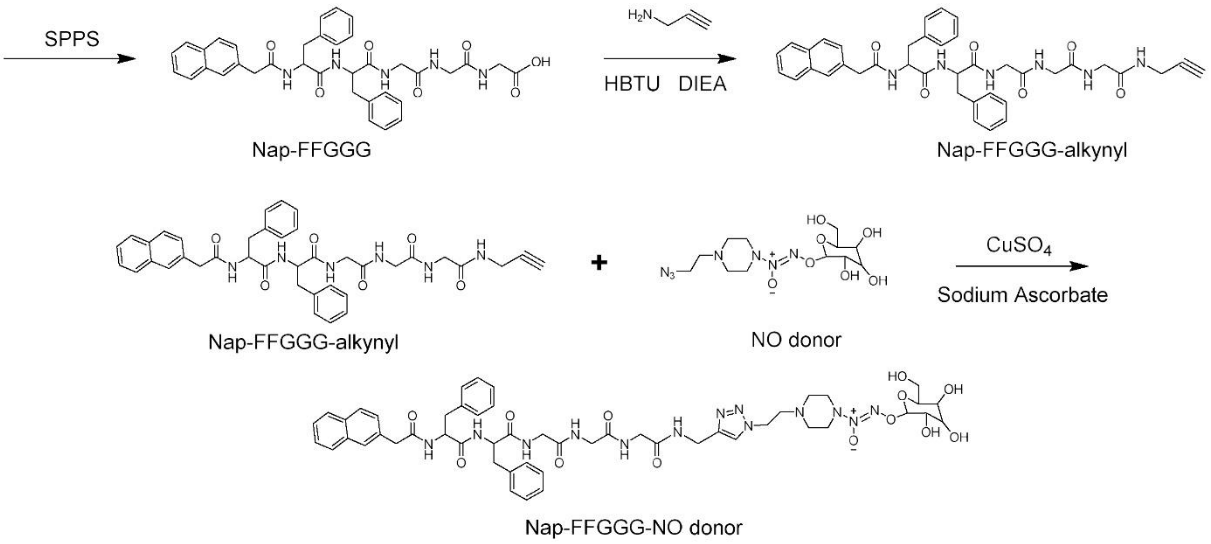

Figure S1. Synthetic pathway for the hydrogelator (NO-peptide conjugate).

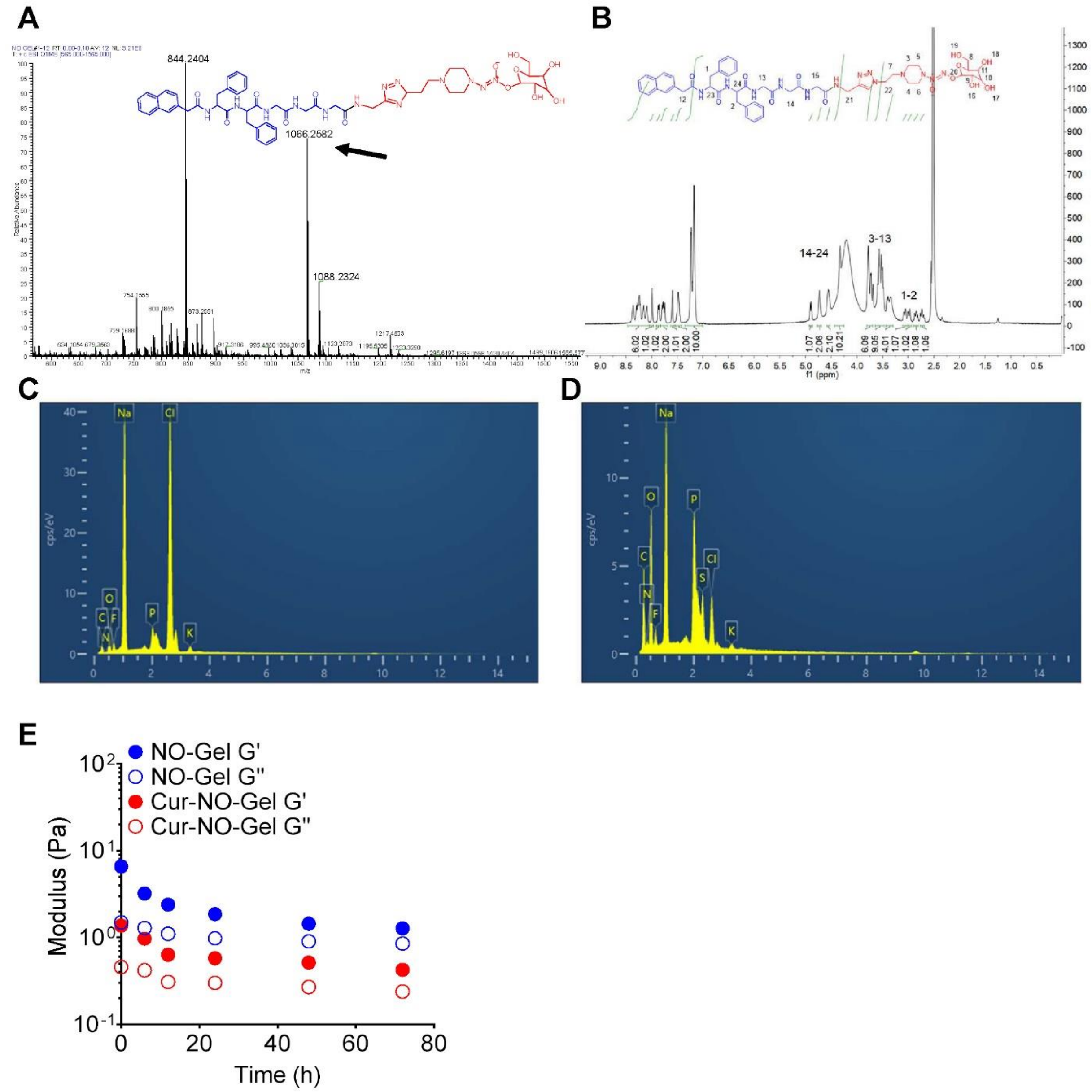

Figure S2. Preparation and characterization of Cur-NO-Gel. A) HR-MS spectra of Nap-FFGGG-NO donor. B) ^{1}H NMR spectra of Nap-FFGGG-NO donor. C) Energy-dispersive X-ray spectroscopy (EDX) analysis of NO-Gel. D) EDX analysis of Cur-NO-Gel. E) Evolution of G' and G'' in the drug release assay (γ = 1 %, ε = 1 rad s^{-1}).

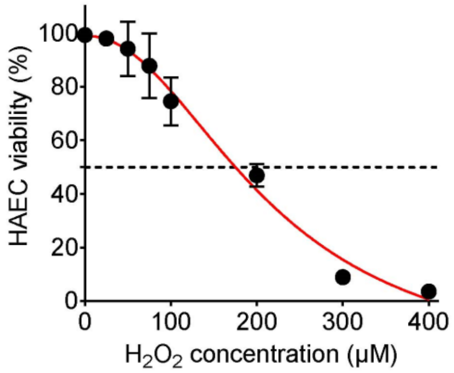

Figure S3 Cell viability of HAECs treated with various concentrations of H_2O_2. Data are shown as mean ± SD.

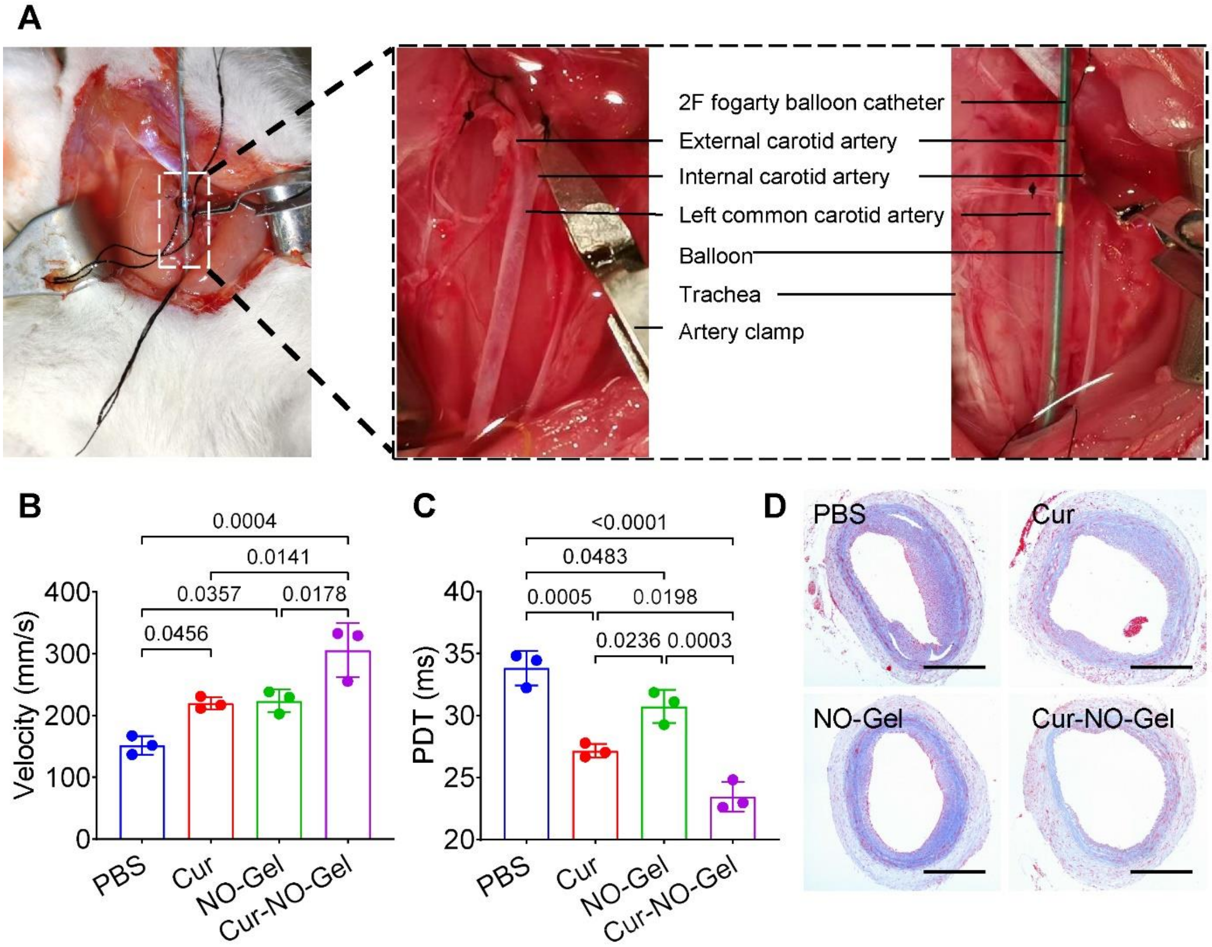

Figure S4. *In vivo* experiments. A) The construction of rat balloon angioplasty model. B) and C) Quantitative analysis of blood velocity and PDT after the indicated treatments (NO donor, 0.15 μmol per rat; Cur dosage, 13 μg per rat). D) Masson-stained sections of rat carotid arteries from rats treated with different compounds. Scale bar: 500 μm. Data are shown as mean ± SD.

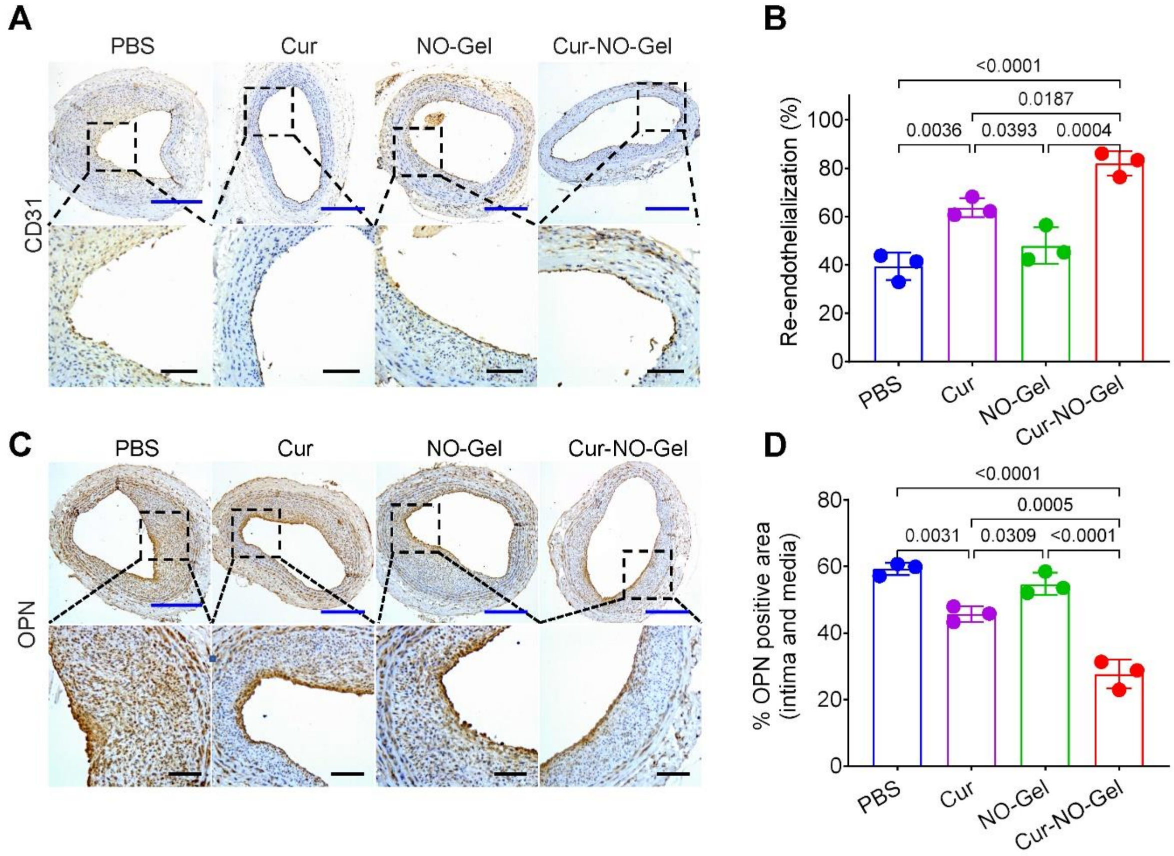

Figure S5. Immunohistochemical staining. A) CD31 immunostaining sections of rat carotid arteries from rats treated with different compounds (NO donor, 0.15 μmol per rat; Cur dosage, 13 μg per rat). Scale bar: 500 μm (blue) and 100 μm (black). B) Quantified data on the percentage of the peri-luminal perimeter that stained positive for CD31staining (n = 3). C) OPN immunostaining sections of carotid arteries in different groups. Scale bar: 500 μm (blue) and 100 μm (black). D) Quantitation of OPN positive area (intima and media) (n = 3). Data are shown as mean ± SD.

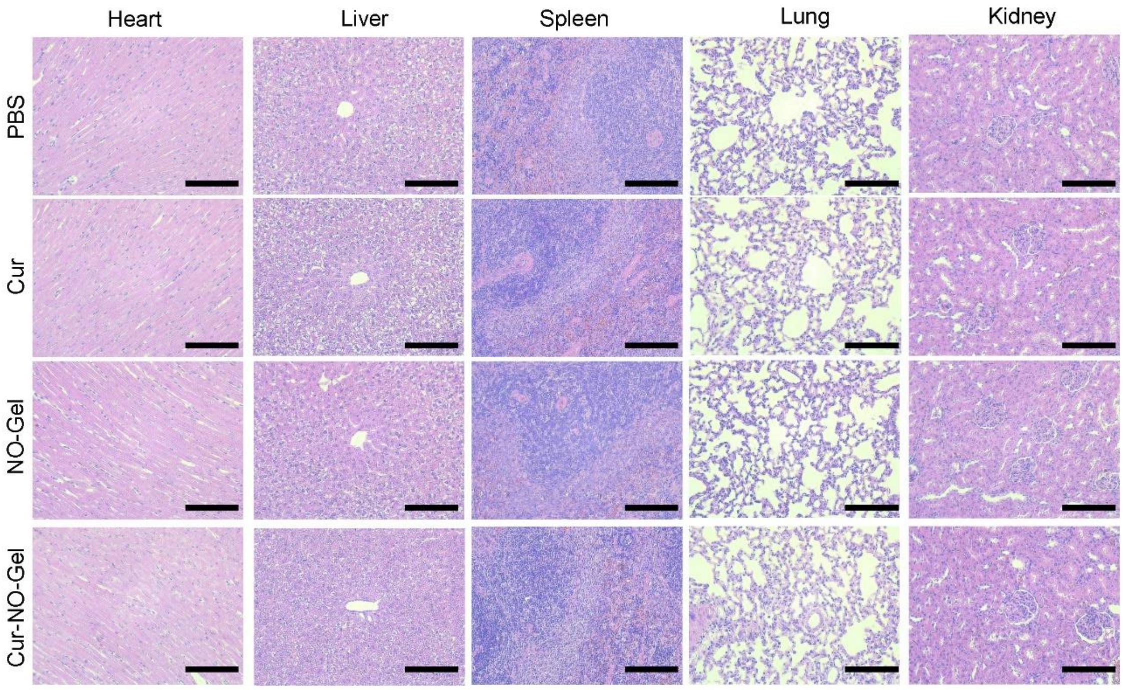

Figure S6. H&E staining of main organs after treatment. Sections of heart, liver, spleen, lung, and kidney were collected to conduct H&E staining (n = 5). Scale bar: 500 nm.

Table S1. The performance differences between Cur-NO-Gel and related drug-eluting stents

	**Drug release**	**Route of administration**	**Reendothelialization**	**Neointima**	**Vasodilatation**
Cur-NO-Gel	Enzyme-triggered NO release	Periadventitial injection	Acceleration	Inhibition	Effective
Paclitaxel-eluting stent[a)]	Uncontrollable	Within the artery lumen	Inhibition	Inhibition	Almost ineffective
Caffeic acid-eluting stent[b)]	ROS-triggered NO release	Within the artery lumen	Acceleration	Almost ineffective	Almost ineffective

[a)]Drug-eluting stent used in clinic; [b)]Drug-eluting stent reported previously (*Advanced Functional Materials* 2023, 33, 2213993.).

Table S2. The impact of different components of hydrogels on stenosis after angioplasty

**Groups**	**Viability of HAECs incubated with H_2O_2**	**Viability of HVSMCs**	**Contractile-phenotype transition**	**Viability of HBVAFs**	**Myofibroblast transformation**	**Reendothelialization**	**Intima/ media area**	**Collagen area**	**Lumen area/ vascular area**
PBS	70.73%	99.76%	1.07	98.48%	1.01	3.86%	1.67	97.57%	0.19
Cur	82.83%	37.82%	1.66	48.71%	0.22	33.87%	0.67	73.57%	0.31
NO-Gel	71.03%	98.39%	1.09	98.03%	1.07	8.78%	1.55	90.56%	0.30
Cur-NO-Gel	77.04%	75.30%	1.39	76.76%	0.74	52.85%	0.28	54.57%	0.38
